# Type I Interferons as Contextual Regulators of B-Cell Tolerance in Type 1 Diabetes

**DOI:** 10.3390/biom16040563

**Published:** 2026-04-10

**Authors:** Mebrahtu G. Tedla, Jamie L. Felton

**Affiliations:** 1 Division of Pediatric Endocrinology, Department of Pediatrics, Indiana University School of Medicine, Indianapolis, IN 46202, USA; mtedla@iu.edu; 2 Herman B Wells Center for Pediatric Research, Department of Pediatrics, Indiana University School of Medicine, Indianapolis, IN 46202, USA; 3 Center for Diabetes and Metabolic Diseases, Indiana University School of Medicine, Indianapolis, IN 46202, USA

**Keywords:** type 1 diabetes, type I interferons, B cells, tolerance, immunotherapy

## Abstract

Type 1 diabetes (T1D) is an immune-mediated disease characterized by progressive autoimmune destruction of pancreatic β cells. Although traditionally viewed as primarily T-cell-driven, B cells play essential roles in disease pathogenesis. In addition to producing islet autoantibodies, B cells contribute to immune activation through antigen presentation and cytokine secretion, thereby shaping autoreactive T-cell responses. The earliest clinical predictor of T1D is the appearance of islet autoantibodies in the blood, reflecting a breach in B-cell tolerance well before symptomatic disease onset. In individuals at high genetic risk, type I interferon (IFN) signatures are detectable in peripheral blood prior to seroconversion, suggesting that type I IFNs may act as upstream regulators of B-cell tolerance. Peripheral tolerance is enforced through layered checkpoints including transitional selection, maintenance of anergy, germinal center regulation, and regulatory B-cell differentiation. Studies in systemic autoimmunity demonstrate that type I IFN signaling lowers B-cell activation thresholds, enhances BCR and TLR responsiveness, promotes survival of autoreactive transitional clones via BAFF induction, destabilizes anergy, and skews differentiation toward inflammatory phenotypes such as T-bet+ age-associated B cells. Consistent with this model, single-cell transcriptomic and BCR repertoire analyses in T1D reveal clonal expansion and proinflammatory signatures in islet-reactive B cells during the preclinical stage. Together, these findings implicate the IFN–B-cell axis as a potential target for early disease modification.

## 1. Introduction

Type 1 diabetes (T1D) is an immune-mediated disorder that results in the destruction of insulin-producing β cells in the pancreas, ultimately leading to chronic hyperglycemia and lifelong dependence on exogenous insulin [1,2]. Islet autoantibodies appear years before the onset of hyperglycemia and are used to identify high-risk individuals for prevention trials [3,4,5,6]; they also reflect early, pathogenic T–B-cell interactions [7,8]. Teplizumab, an anti-CD3 antibody that targets T cells, was recently approved to delay the onset of T1D in multiple autoantibody-positive individuals [9,10,11]. However, responses to teplizumab are variable, underscoring the multifactorial nature of disease progression [12]. In addition to T cells, B cells play important roles in T1D progression as antibody-producing and antigen-presenting cells [13,14,15,16]. B-cell depletion therapy in recent onset T1D transiently preserves endogenous insulin secretion [17,18], highlighting a critical B-cell contribution to T1D progression.

Normally, self-reactive B cells remain unresponsive, or anergic, in circulation [19]. Islet autoantibody development, termed seroconversion, indicates failure of these tolerance mechanisms. In both non-obese diabetic (NOD) mice and humans, type I interferon (IFN) transcriptional signatures appear in the blood before seroconversion, suggesting that IFN signaling may contribute to B-cell tolerance loss [20,21]. While there is ample literature supporting type I IFN as a key modulator of β cell stress within the islet microenvironment [22,23,24], comparatively little attention has been given to how type I IFNs influence B cells within this context. Studies in other autoimmune conditions demonstrate that type I IFNs can lower B-cell activation thresholds, alter B-cell fate decisions, and promote survival and differentiation of autoreactive B cells [25,26]. In this review, we synthesize emerging human and experimental data to propose that type I IFNs act as contextual regulators of B-cell tolerance in T1D.

## 2. Peripheral B-Cell Tolerance as a Dynamic Regulatory System

During B-cell development in the bone marrow, hematopoietic stem cells differentiate through sequential stages marked by rearrangement of B-cell receptor (BCR) genes. This random, somatic recombination process, termed V(D)J recombination, generates a highly diverse BCR repertoire capable of recognizing a wide array of antigens [27,28]. Because V(D)J recombination is stochastic, some BCRs will inevitably recognize self; in fact, up to 75% of BCRs on early, immature B cells in the bone marrow are autoreactive [29]. Central tolerance mechanisms—including clonal deletion, receptor editing, and developmental arrest—eliminate or silence many strongly self-reactive clones [27,30]. However, this process is intentionally incomplete. Some low affinity autoreactive B cells exit the bone marrow, reflecting a delicate balance between maintaining self-tolerance while preserving sufficient repertoire diversity to recognize pathogens. For this reason, autoreactive B cells are common in the mature repertoire and are typically controlled by peripheral tolerance checkpoints rather than eliminated. Peripheral tolerance, therefore, functions as a dynamic, multilayered system that restrains autoreactive clones after they enter the mature repertoire [31].

### 2.1. Transitional B-Cell Decision Point: Survival of Autoreactive Clones

Upon entering the spleen, transitional B cells undergo a regulated selection process that determines whether they die, become anergic, or mature into naïve B cells [32,33,34]. Transitional B cells are highly sensitive to BCR signaling—strong engagement with self-antigen induces apoptosis, whereas chronic low-level recognition promotes anergy. Cells receiving appropriate low-level “tonic” BCR signaling progress to the later transitional stages, where survival depends on access to limited B-cell activating factor (BAFF) [35]. Competition for BAFF acts as a key checkpoint, allowing cells with favorable signaling profiles to mature. Additional cues—including Notch2 signaling, antigen availability, and positioning within the splenic microenvironment—guide commitment toward follicular or marginal zone fates. Thus, splenic entry is a critical checkpoint at which BCR signal strength, survival factors, and environmental context determine whether autoreactive clones are eliminated, silenced, or incorporated into the mature repertoire.

### 2.2. Anergy: Persistent Functional Silencing

In the mature repertoire, autoreactive B cells are primarily controlled through functional restraint rather than deletion. Anergy is the dominant mechanism, arising from chronic low-level self-antigen engagement without adequate co-stimulation [36,37,38]. Anergic B cells exhibit reduced surface IgM, dampened BCR signaling, impaired calcium flux, shorted lifespan, and exclusion from follicular niches. This hyporesponsive state is actively maintained by inhibitory pathways involving phosphatases, such as SHP-1 and PTEN, and receptors, such as FcγRIIB and CD22 [39,40,41,42]. Importantly, anergy is reversible; inflammatory signals, including TLR ligation or excessive BAFF, can restore responsiveness and promote differentiation [43,44]. Anergy, therefore, is not passive ignorance, but an actively maintained, functionally silenced state [38].

### 2.3. Cell Fate Decisions: Regulatory B-Cell Differentiation

Some mature B cells acquire regulatory functions. Regulatory B cells (Bregs), defined functionally by their production of IL-10 and other immunomodulatory cytokines, suppress effector T-cell responses and support Treg stability [45,46]. They arise from transitional or mature pools, following antigen encounter combined with inflammatory or innate signals (e.g., CD40, TLRs). Unlike anergy, which reflects insufficient co-stimulation, Breg differentiation requires integrated antigenic and inflammatory cues that actively program immunoregulation. Thus, the balance between tolerance and regulation is shaped by the broader cytokine and innate milieu in which B cells encounter antigen [47].

### 2.4. Additional Mechanisms

Mature B-cell tolerance is further reinforced by extrinsic checkpoints that maintain high activation thresholds for autoreactive clones. A central safeguard is dependence on cognate CD4+ T-cell help: autoreactive B cells typically fail to receive CD40-CD40L interactions and T-follicular-helper (Tfh)-derived cytokines such as IL-21, limiting their entry into germinal centers and preventing affinity maturation or plasma cell differentiation [48,49]. Inhibitory receptors such as FcγRIIB, CD22, and PD-1 ligand interactions further dampen BCR signaling, especially in response to self-antigen-containing immune complexes [48,50]. Finally, germinal center selection eliminates B cells that acquire or retain autoreactivity during somatic hypermutation, thereby preventing the generation of high-affinity autoantibodies [48].

## 3. Lessons from Other Autoimmune Diseases: Type I IFNs as Regulators of B-Cell Tolerance

Studies in systemic autoimmune diseases, such as systemic lupus erythematous (SLE) and Sjogren’s syndrome, provide compelling evidence that type I IFNs regulate multiple layers of B-cell tolerance [51]. Type I IFNs shape activation thresholds, survival, and cell-fate decisions that determine whether autoreactive B cells remain functionally silent or become pathogenic [25]. These findings provide a framework for understanding how type I IFNs could alter tolerance checkpoints in organ-specific autoimmunity, including T1D.

### 3.1. Type I IFNs and Survival of Autoreactive Clones

Following bone marrow egress, transitional B cells enter the spleen, a key site of fate determination. In autoimmune settings, type I IFNs influence both intrinsic and extrinsic mechanisms governing their survival. In lupus-prone mice, B-cell-derived IFN-β enhances transitional B-cell survival and responsiveness to BCR/TLR signals, shifting selection toward survival rather than deletion [51]. In human studies, plasma from SLE patients supports increased transitional B-cell survival in a type I IFN-dependent manner. Direct treatment with IFN-α enhanced transitional B-cell survival and pro-inflammatory functions, tying systemic IFN overactivation to altered transitional B-cell tolerance in human disease [26]. Intriguingly, in T1D, anti-insulin B cells have been shown to accumulate in late transitional and marginal zone compartments [52], though whether this developmental trait is driven or dependent on IFN signaling has not been directly tested.

Type I IFNs also indirectly enhance survival by increasing BAFF production from myeloid and stromal cells. Elevated BAFF relaxes selection thresholds, allowing autoreactive transitional B cells to persist and enter the mature repertoire, as demonstrated in BAFF transgenic mouse models [53]. In Sjogren’s syndrome patients, type I IFN signatures in monocytes correlate with increased BAFF expression, linking systemic type I IFN activity with elevated BAFF production in myeloid cells. Conversely, in lupus models, BAFF blockade reduces autoreactive B-cell survival and ameliorates disease, underscoring the importance of this axis [54]. Thus, IFN-driven BAFF production represents a B-cell extrinsic mechanism by which inflammatory context reshapes peripheral selection pressure.

### 3.2. Type I IFNs and Anergy

Anergy is an actively maintained hyporesponsive state [38]; however, accumulating evidence from mouse models and human autoimmune disease demonstrates that type I IFNs can destabilize it. Type I IFNs signal through IFNAR, activating JAK1 and TYK2 and inducing STAT1/STAT2 phosphorylation with formation of the ISGF3 complex (STAT1/STAT2/IRF9), which drives transcription of interferon-stimulated genes (ISGs). In lupus-prone models, B-cell-intrinsic IFNAR signaling is required for the loss of tolerance and development of autoreactive B cells [55]. IFNAR signaling alters transcriptional programs governing BCR responsiveness, antigen presentation, and co-stimulatory molecule expression, effectively lowering activation thresholds of autoreactive clones. Mechanistically, type I IFNs enhance expression of TLR7 and related pathways, increasing responsiveness to nucleic acid-containing self-antigens [56,57]. STAT1 and IRF9 are required for optimal autoantibody production and B-cell activation in lupus models, linking canonical IFN signaling pathways directly to autoreactive B-cell differentiation [58]. These data support a model in which type I IFN signaling potentiates BCR and TLR co-engagement, overcoming the signaling attenuation that characterizes anergy. Evidence that loss of B-cell anergy contributes to the development of T1D has been established by Smith et al. Specifically, they demonstrated that high-affinity insulin-binding B cells, normally present within the anergic B-cell compartment in healthy individuals, are absent both prior to and at the time of T1D diagnosis [59]. This loss has been attributed to an as-yet unidentified environmental trigger, raising the possibility that type I IFNs may play a role.

Type I IFNs further counter anergy by increasing MHC class II and co-stimulatory molecule expression on B cells, enhancing access to T-cell help [25], and promoting differentiation toward extrafollicular plasmablast and inflammatory T-bet+ CD11c+ “age-associated” or autoimmune-associated B cells (ABCs) [60]. Consistent with this, SLE patients exhibit a strong interferon signature in peripheral blood that correlates with autoantibody levels and disease activity [61,62]. Importantly, transitional and naive B-cell subsets from SLE patients display heightened ISG expression and enhanced survival responses to IFN-α [54], consistent with relaxation of peripheral tolerance checkpoints. Together, these findings indicate that type I IFNs undermine B-cell anergy through coordinated transcriptional reprogramming, amplification of innate co-stimulatory pathways, and enhanced access to T-cell help, thereby reversing anergy to promote tolerance loss. In T1D, IFN signatures have been detected in the peripheral blood of high-risk individuals as well as some autoantibody-negative first-degree relatives prior to disease onset [63]. However, whether the presence, magnitude, or persistence of this signature predicts progression, analogous to its role in SLE, remains unclear. Defining the prognostic and mechanistic significance of IFN responses in early T1D represents an important opportunity for risk stratification and biomarker development.

### 3.3. Type I IFNs and Regulatory B-Cell Development

Although type I IFNs are best known for promoting pro-inflammatory B-cell activation, accumulating evidence indicates that type I IFN-rich environments also influence the development and function of Bregs, most commonly defined by IL-10 production [64]. Breg differentiation requires integration of BCR, CD40, TLR, and cytokine signals that converge on STAT3- and c-Maf-dependent transcriptional programs driving IL-10 expression [46,65]. By reshaping TLR responsiveness and antigen presentation, type I IFNs alter this signaling context. In autoimmune diseases characterized by strong IFN signatures, such as SLE, B cells exhibit heightened ISG expression alongside impaired or unstable regulatory phenotypes, suggesting that chronic IFN exposure skews B-cell differentiation toward inflammatory fates [54,60]. In NOD mice, transient blockade of BAFF, whose levels can be elevated by type I IFNs, prevents development of T1D by promoting the expansion of Bregs [66], suggesting a potentially similar role in human disease. Thus, type I IFNs not only promote activation but broadly reprogram B-cell fate, shifting the balance from regulation toward pathogenic immunity.

## 4. Beyond the β Cell: Type I IFN and B-Cell Crosstalk in T1D

In T1D, type I IFNs have also been identified as central mediators of disease pathogenesis, particularly in the earliest stages of islet autoimmunity. Longitudinal studies in genetically at-risk children demonstrate that a peripheral IFN transcriptional signature precedes seroconversion and clinical onset [63], and pancreatic islets from individuals with recent onset T1D display marked HLA class I hyperexpression and induction of ISGs, consistent with local IFN exposure [67,68]. Mechanistic studies in human β cells have shown that IFN-α enhances antigen presentation, induces chemokine production such as CXCL10, and promotes cellular stress pathways that increase β-cell visibility to autoreactive CD8+ T cells [69,70,71]. Consequently, the prevailing model has focused largely on type I IFN-mediated amplification of β cell immunogenicity. However, type I IFNs are not β-cell-restricted mediators; they are systemic cytokines with profound effects on adaptive immune compartments, as demonstrated by their roles in other systemic autoimmune diseases.

**Figure 1:**
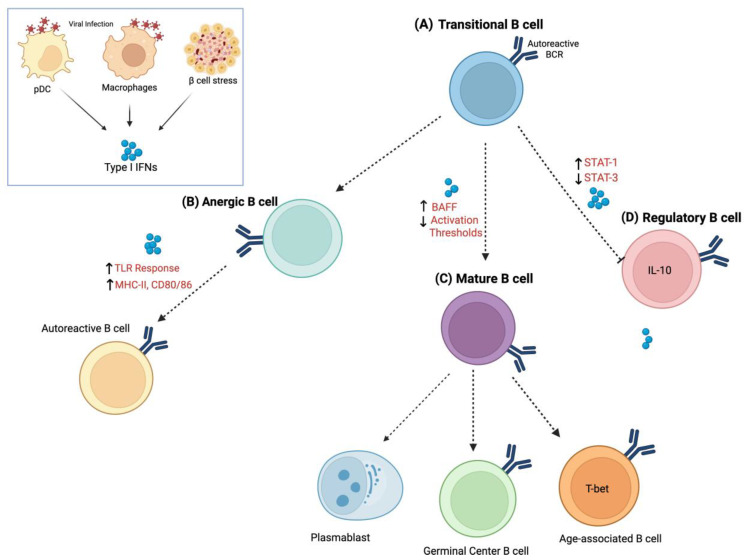
Type I interferon-mediated reprogramming of the B-cell compartment in type 1 diabetes. Type I IFNs, induced by viral infection, innate immune activation, or β-cell stress, function as upstream regulators of B-cell tolerance checkpoints in T1D. (A) At the transitional checkpoint, type I IFN-induced BAFF production and enhanced tonic signaling relax competitive survival constraints, permitting autoreactive clones to persist. (B) Within the mature compartment, type I IFNs destabilize anergy by enhancing antigen presentation, upregulating co-stimulatory molecules, and amplifying innate co-signals, thereby restoring responsiveness of previously silenced islet-reactive B cells. (C) Concurrently, type I IFNs skew differentiation of mature naïve B cells toward inflammatory fates, including T-bet+ age-associated B cells, extrafollicular plasmablasts, and germinal center B cells, promoting clonal expansion and autoantibody production. (D) In parallel, chronic type I IFN exposure may impair IL-10-producing Breg development by favoring STAT1-dominant programs over STAT3-dependent regulatory pathways, weakening immune restraint. While direct examination of the effect of type I IFNs on B cells in T1D is sparse, there are several studies that support type I IFN-driven loss of tolerance for B in this context. Williams et al. [76] generated a site-directed anti-insulin heavy chain knock-in mouse (VH125SD) to examine peripheral tolerance in class-switch competent autoreactive B cells. Although anti-insulin B cells entered the mature follicular repertoire and were maintained in an anergic state—failing to produce IgG following T-cell-dependent immunization—linked BCR and TLR4 engagement using an insulin–Brucella abortus conjugate reversed anergy, restored surface IgM expression, induced germinal center formation, and drove IgG2a autoantibody production [74]. Importantly, autoreactive clones from the pre-immune repertoire were recruited into germinal centers in a largely T-cell-independent manner. These findings demonstrate that innate co-stimulatory signals can override peripheral B-cell tolerance checkpoints, providing a mechanistic framework by which the IFN-rich inflammatory environment observed prior to seroconversion in T1D could destabilize anergy and promote pathogenic B-cell differentiation. Created in Biorender. Jamie L. Felton (2026).

In addition to autoantibody production, B cells play important roles in T1D progression through antigen presentation, T-cell priming, and cytokine-mediated immune regulation [72,73,74]. In the NOD mouse, B-cell deficiency markedly reduces diabetes incidence, underscoring their functional importance beyond serologic biomarkers [59]. In humans, B-cell depletion with rituximab delays decline of endogenous insulin secretion in recent-onset T1D, further implicating B cells in disease progression [17,18]. In addition to the peripheral IFN signature, the period before seroconversion is marked by the absence of an anergic subset of insulin-binding B cells [59,75]. Within this context, type I IFNs represent a compelling potential upstream signal capable of reshaping B-cell biology and driving tolerance loss in T1D. While direct evidence in T1D remains limited, studies in systemic autoimmune disease support a model that can be extrapolated to T1D based on these defined B-cell roles, in which type I IFNs reshape B-cell tolerance by promoting survival, lowering activation thresholds, and skewing B-cell fate toward pathogenic outcomes. Defining how type I IFNs shape the B-cell compartment in T1D may therefore reveal critical links between innate pathways and adaptive tolerance breakdown (Figure 1).

Recent single-cell analyses of islet antigen-reactive (IAR) B cells in individuals across the T1D continuum provide direct human evidence of progressive B-cell tolerance destabilization in T1D. Nicholas et al. [77] demonstrated that IAR B cells from autoantibody-positive and recent-onset T1D donors exhibit enhanced BCR-signaling signatures (BLNK, SYK, CD79B), reduced expression of inhibitory regulators such as LYN, increased antigen presentation machinery (CIITA, CD40, HLA genes), and enrichment of proinflammatory and infection-associated transcriptional programs [77]. These molecular features are consistent with erosion of peripheral anergy, a state normally maintained by attenuated BCR signaling and tonic inhibitory feedback. The expansion of polyreactive clones and increased BCR repertoire diversity in autoantibody-positive donors further suggests that early tolerance checkpoints—including transitional selection and maintenance of anergic naïve pools may be relaxed, permitting autoreactive clones to persist and expand. Notably, clonally expanded IAR B cells were enriched within antigen-experienced subsets, including ABCs and plasmablasts, consistent with skewing toward inflammatory differentiation pathways that may bypass stringent germinal center regulation. When considered alongside the well-established type I IFN signature present prior to seroconversion in T1D, these findings support a unified model in which type I IFN amplifies BCR and innate signaling, lowers activation thresholds at transitional and mature stages, destabilizes anergy, and promotes extrafollicular differentiation of autoreactive B cells. In this framework, IFN-driven inflammatory cues do not merely enhance β-cell immunogenicity but actively reshape the B-cell compartment, facilitating persistence, clonal expansion, and functional reprogramming of islet-reactive clones prior to early T1D progression.

In addition to promoting inflammatory B-cell activation, alterations in the Breg compartment likely contribute to tolerance breakdown in T1D. In individuals with T1D, several studies have reported reduced frequencies and/or impaired suppressive function of IL-10-producing B cells, particularly within transitional and memory subsets [78,79], suggesting compromised regulatory restraint during disease progression. An IFN-rich inflammatory environment may further skew this balance. Type I IFNs activate JAK/STAT pathways with strong STAT1 bias, enhance TLR responsiveness, and promote differentiation toward pro-inflammatory phenotypes such as T bet+ ABCs and plasmablasts [55,60]. In contrast, IL-10 production and regulatory B-cell programming depend in part on STAT3-driven transcriptional networks [80]. We hypothesize, therefore, that chronic type I IFN exposure could antagonize STAT3-dependent regulatory pathways while favoring inflammatory differentiation, simultaneously destabilizing anergy and impairing Breg development or function. In this model, type I IFNs not only amplify autoreactive B-cell activation but also erode regulatory counterbalances within the B-cell compartment.

At the same time, T1D-associated genetic susceptibility loci provide strong support for a central role of type I IFN biology in disease pathogenesis, likely secondary to intrinsic effects on B cells, specifically. *IFIH1* (which encodes MDA5) is an anti-viral cytosolic RNA sensor that triggers type I IFN production, and its association with T1D risk has been attributed to exacerbated anti-viral responses [81]. Expression of its risk variant in NOD mice led to enhanced ISG signatures, increased plasma cell frequency, and accelerated T1D [82]. Polymorphisms in *TYK2* have also been associated with increased T1D risk, thought to be primarily secondary to enhanced antigen presentation by stimulating MHC class I expression in the β cell, leading to T-cell activation and recruitment to pancreatic islets [83]. However, *TYK2* has also been shown to modulate splenic B-cell development, and its deletion increased ABC development in mouse models [84]. *PTPN22*, which encodes the protein tyrosine phosphatase Lyp, carries the highest genetic risk after HLA Class II and *INS*. It is a known negative regulator of T-cell receptor signaling and modulator of type I IFN production in macrophages and dendritic cells [85]; however, *PTPN22* risk variants have also been shown to alter BCR signal transduction [86] and enhance positive selection of autoreactive B cells at distinct tolerance checkpoints in mouse models, highlighting its role in shaping the B-cell repertoire [87]. Taken together, it is plausible that these risk-loci may shift B-cell homeostasis away from anergy and regulation and toward inflammatory phenotypes.

Although there is currently no direct evidence linking type I IFNs to B-cell-tolerance loss in T1D, both human and mouse studies support a broader role for type I IFNs in T1D pathogenesis (summarized in Table 1). This evidence, taken together alongside mechanistic insights from other autoimmune diseases, suggests that type I IFNs have the potential to influence B-cell tolerance in T1D and should be critically assessed in this context.

## 5. Therapeutic Implications: Targeting the IFN–B-Cell Axis in T1D

The emerging model of type I IFN-mediated B-cell reprogramming in T1D has important therapeutic implications. Durable immune modulation in T1D remains an unrealized goal. To date, immune modulation with B-cell-depleting agent rituximab has resulted in only transient preservation of endogenous insulin secretion [17,18]. Post hoc analyses of this trial demonstrated that, following B-cell depletion, re-emerging B cells were primarily newly generated transitional and naïve B cells, and that rituximab did not reset defective early B-cell tolerance checkpoints [88]. In parallel, rituximab most strongly suppressed insulin autoantibody production, while effects on anti-GAD, IA-2, and ZnT8 autoantibodies were much smaller and that lower baseline levels of insulin autoantibodies were associated with improved response [89]. Together, these findings suggest that rituximab responders and nonresponders may represent biologically distinct groups rather than a continuum of treatment effect. Whether these groups are defined by increased IFN activity early in the disease remains unknown, but it is especially compelling, given the evidence that IFN signatures precede islet autoimmunity in genetically at-risk children [63].

As small-molecule JAK inhibitors targeting IFN-driven JAK-STAT signaling have gained approval in rheumatologic diseases, clinical trials testing similar agents in new-onset T1D have expanded as well. Notably, the BANDIT trial demonstrated preservation of β-cell function following treatment with the JAK1/2 inhibitor baricitinib in individuals with recent-onset T1D [90], leading to its advancement into a phase-3 trial assessing whether earlier intervention can delay disease progression in at-risk individuals (NCT07222137). In parallel, ongoing studies are evaluating the JAK1-selective inhibitor abrocitinib and the JAK3/TEC family kinase inhibitor ritlecitinib in new-onset T1D (NCT05743244). While these active investigations have shown promise in preserving β-cell function, they are accompanied by the risks inherent to broad inhibition of pleiotropic signaling pathways, including an increased susceptibility to infections. This is especially pertinent in light of the need for long-term, ongoing treatment in presymptomatic individuals.

Focusing more specifically on the IFN–B-cell axis specifically, therefore, may provide an opportunity to refine therapeutic strategies by selectively modulating key B-cell developmental and functional pathways. Because B cells are directly responsive to type I IFNs via JAK-STAT signaling, broad inhibition with JAK kinases would be expected to reduce ISG expression and attenuate IFN-driven B-cell activation, survival, and antigen presentation. In T1D, where B cells play a central role in antigen presentation and in shaping T-cell responses, IFN signaling may act as a key regulator of B-cell function. Therapeutically targeting this pathway may therefore not only dampen inflammation but also reprogram B-cell fate decisions toward tolerance-promoting states. Importantly, human single-cell transcriptomic and BCR repertoire data indicating that autoreactive B-cell expansion and activation are most pronounced in the autoantibody-positive stage [9] suggest that intervention during this preclinical window may be particularly impactful.

Upstream of these processes, viral sensing pathways that drive IFN production, particularly those mediated by MDA5 (encoded by *IFIH1*), a known T1D susceptibility locus, represent an additional layer of regulation that may shape the IFN milieu in early disease. Infection by the enterovirus, coxsackie B, has been associated with the onset of islet autoimmunity [91,92], and dysregulated activation of these pathways could contribute to the IFN-rich environment that conditions B-cell activation and lowers tolerance thresholds. Although therapeutic targeting of these upstream pathways remains largely theoretical in T1D, modulation at this level could, in principle, preventthe initiation of IFN-driven immune dysregulation that precedes overt autoimmunity. However, given the essential role of these pathways in antiviral immunity, strategies aimed at selectively attenuating pathogenic IFN responses without compromising host defense will be critical.

The presence of an early type I IFN signature in T1D suggests that targeting IFN signaling during this window may preferentially impact B cells. Strategies aimed at restoring Breg function or maintaining B-cell anergy, potentially through STAT-3 supportive or BAFF-modulating approaches, may selectively influence autoreactive B-cell populations at this stage, offering an opportunity for targeted intervention rather than broad B-cell depletion. Complementary approaches could include IFNAR blockade, inhibition of downstream JAK-STAT signaling, or selective targeting of TLR7/9 pathways that synergize with type I IFNs to amplify autoreactive B-cell activation. Together, these insights support a shift in perspective from viewing B cells solely as antibody producers to recognizing them as dynamic participants in IFN-driven immune dysregulation, highlighting the IFN–B-cell axis as a rational target for disease-modifying therapies in early T1D.

## 6. Conclusions and Knowledge Gaps

Early T1D is characterized by a prominent type I IFN transcriptional signature and a concurrent loss of anergic B cells, highlighting the disruption of B-cell tolerance as a central feature of disease pathogenesis. The convergence of mechanistic studies, human single-cell transcriptomics, and BCR repertoire analyses supports a model in which type I IFNs act as upstream orchestrators of B-cell tolerance breakdown. By lowering activation thresholds at transitional and mature checkpoints, destabilizing anergy, amplifying innate co-stimulatory pathways, skewing differentiation toward inflammatory effector states, and potentially impairing Breg programs, type I IFNs are well positioned to reshape the B-cell compartment in ways that favor autoreactive persistence and expansion. Notably, many of these alterations are most pronounced in the preclinical, autoantibody-positive stage, suggesting that IFN-driven B-cell reprogramming is an early and potentially reversible event in disease pathogenesis.

Despite these advances, critical knowledge gaps remain: while a type I IFN transcriptional signature and the loss of anergic B cells are each well documented in early T1D, the mechanistic and temporal relationship between these phenomena has not yet been defined. It is unclear whether IFN signaling directly drives the erosion of B-cell anergy, acts indirectly through other immune compartments, or represents a parallel process triggered by a shared upstream event. Future work should focus on defining the temporal sequence of IFN exposure, B-cell tolerance erosion, and clonal expansion in at-risk individuals, as well as identifying biomarkers that capture this transition. A deeper understanding of the IFN–B-cell axis may ultimately enable more precise, stage-specific interventions that restore immune tolerance before irreversible β-cell destruction occurs.

## Figures and Tables

**Table 1:** Evidence for type I IFN roles in autoimmune diseases and T1D.

	Study Category	Disease/Model	Experimental Context	Key Finding Relevant to Type I IFN	Reference
T1D	Animal	NOD mouse	IFN-α induction, IFNAR1 blockade	Type I IFN accelerates T1D	[79]
		NOD mouse	Anti-IFNAR1; pDC depletion	Type I IFN pathway blockade prevents/delays T1D	[80]
		NOD IFNAR1^−/−^	Genetic IFNAR deficiency	Reduced ISG expression without full protection	[81]
		NOD (adult prediabetic)	IFNAR blockade timing	Late Type I IFN blockade accelerates disease & enhances Th1 responses	[82]
		RIP-LCMV viral model	Cell-specific IFNAR deletion	Macrophage Type I IFN promotes diabetogenic CD8 T cells	[19]
		STZ-induced diabetes	IFN pathway inhibition	Type I IFN contributes to immune dysregulation	[83]
	Human	At-risk children BABYDIET / DIPP cohorts	Longitudinal study/transcriptomics	Type I IFN signature precedes islet autoimmunity	[63,84]
		New-onset T1D	pDC frequency & IFN-α production	Increased IFN-α producing pDCs in T1D	[85]
Other diseases	Animal	SLE (murine)	B cell intrinsic IFNAR deletion	Type I IFN signaling within B cells promotes autoreactive GC and AFC differentiation	[54]
		SLE (pristane-induced)	IFNAR2 regulation of TLR7/9	Type I IFN enhances B-cell responsiveness to nucleic-acid autoantigens	[86]
		Sjögren (NOD-derived model)	IFN signaling and B-cell phenotype	Type I IFN shapes pathogenic B-cell phenotypes	[87]
	Human	SLE	B-cell tetherin IFN biomarker	B-cell Type I IFN response correlates with disease activity	[88]
		SLE	Leukocyte-specific IFN signatures	Type I IFN programs detectable across immune subsets	[89]
		Sjogren’s syndrome (SS)	Type I IFNs signatures	Systemic Type I IFN linked to B-cell hyperactivity	[90]
		Sjogren’s syndrome (SS)	Single-cell B-cell profiling	Type I IFN associated programs define pathogenic B-cell states	[91]

## Data Availability

Data sharing is not applicable. No new data were created or analyzed in this study.
